# In-situ x-ray fluorescence imaging of the endogenous iodine distribution in murine thyroids

**DOI:** 10.1038/s41598-022-06786-4

**Published:** 2022-02-21

**Authors:** Christian Körnig, Theresa Staufer, Oliver Schmutzler, Tanja Bedke, Andres Machicote, Beibei Liu, Yang Liu, Elisabetta Gargioni, Neus Feliu, Wolfgang J. Parak, Samuel Huber, Florian Grüner

**Affiliations:** 1 Fachbereich Physik, Universität Hamburg and Center for Free-Electron Laser Science (CFEL), Luruper Chaussee 149, 22761 Hamburg, Germany; 2 I. Department of Medicine, Hamburg Center for Translational Immunology (HCTI), University Medical Center Hamburg-Eppendorf, 20246 Hamburg, Germany; 3 Fachbereich Physik, Universität Hamburg and Center for Hybrid Nanostructures (CHyN), Luruper Chaussee 149, 22761 Hamburg, Germany; 4 Department of Radiotherapy and Radiation Oncology, University Medical Center Hamburg-Eppendorf, Martinistraße 52, 20246 Hamburg, Germany; 5 Fraunhofer Center for Applied Nanotechnology (CAN), Grindelallee 117, Hamburg, Germany

**Keywords:** Imaging techniques, Nanomedicine, Preclinical research

## Abstract

X-ray fluorescence imaging (XFI) is a non-invasive detection method of small quantities of elements, which can be excited to emit fluorescence x-ray photons upon irradiation with an incident x-ray beam. In particular, it can be used to measure nanoparticle uptake in cells and tissue, thus making it a versatile medical imaging modality. However, due to substantially increased multiple Compton scattering background in the measured x-ray spectra, its sensitivity severely decreases for thicker objects, so far limiting its applicability for tracking very small quantities under in-vivo conditions. Reducing the detection limit would enable the ability to track labeled cells, promising new insights into immune response and pharmacokinetics. We present a synchrotron-based approach for reducing the minimal detectable marker concentration by demonstrating the feasibility of XFI for measuring the yet inaccessible distribution of the endogenous iodine in murine thyroids under in-vivo conform conditions. This result can be used as a reference case for the design of future preclinical XFI applications as mentioned above.

## Introduction

X-ray fluorescence analysis (XRF) is a powerful tool for quantitative elemental mass determination of trace elements. In contrast to alternative methods like inductively coupled plasma mass spectrometry (ICP-MS), XRF is non-destructive (neglecting the radiation damage) and does only require minimal sample preparation. In combination with the advance in the field of nanoparticles in recent years, XRF is a promising tool for preclinical cell tracking, nanoparticle uptake or drug delivery studies^1–8^. In particular, it enables cell uptake measurements of medium and high-Z elements down to the pg/cell level^3,4,9^. However, when translating this technique from in-vitro to in-vivo, the increased sample thickness imposes a major challenge for separating the fluorescence signal from the multiple Compton scattering background. This limits the sensitivity of XRF in the application of biomedical imaging, referred to as x-ray fluorescence imaging (XFI). In recent years, several techniques have been presented to overcome this problem^10–14^, even showing the feasibility of in-vivo XFI in humans^12^. Additionally, first in-vivo XFI measurements of mice have been performed, however, so far limited to the use of high concentrations of nanoparticles injected into the tail vein^13–15^. Typically, x-ray tubes are used as the fluorescence excitation source and a pinhole collimator combined with a detector array forming a camera obscura geometry provide a spatially resolved fluorescence image^13,14^. By using a pinhole imaging geometry, 3D information can be obtained without the need of rotation, but at the cost of dramatically reducing the detectable solid angle and therefore sensitivity. Alternatively, a multilayer mirror has been proposed^11,15^, to generate a “spectrally-matched” quasi monoenergetic pin beam from a liquid metal jet x-ray source. With this setup, the accumulation of 1 mg molybdenum nanoparticles in the liver of mice has been tracked in-vivo. This method requires the use of tracer elements with K-edges close to the Indium K_α_ emission line at 24 keV as incident energy. This leaves only a small selection of elements with fluorescence energies large enough to have sufficient transmission through the tissue, low enough to be excitable by the source and chemical properties to allow synthesis of non-toxic nanoparticles^1^.

To be applicable to cell tracking studies, where the amount of fluorescence marker is limited by the uptake per cell and maximum number of injected cells and therefore the total fluorescence element mass is expected to be in the order of µg^2,16–19^, the sensitivity of XFI has to be improved by about 2–3 orders of magnitude. Additionally, a broader usable fluorescence energy range is desirable to allow for different marker elements and will ultimately be needed for multi-element tracing^1^. To address these issues, a high flux and high energy monoenergetic x-ray source is needed.

In this work, we present a synchrotron-based measurement of the endogenous thyroidal iodine mass in wildtype and Rag1-deficient C57BL/6 mice using x-ray fluorescence imaging to demonstrate the detection of small quantities of iodine. Previous studies based on single photon emission computed tomography (SPECT) and scintigraphy rely on the use of radiotracers—typically between 10 and 300 µCi (0.4 to 11 MBq) ^125^I or ^131^I^20–24^ or even exceeding 800 µCi (30 MBq)^25^ and have only been able to detect uptake rates of iodine radiotracer^26,27^. Local radiation dose in rats were determined to be 2.6 Gy/MBq for ^125^I and 21 Gy/MBq for ^131^I and doses in mice are expected to be even higher due to the smaller volume^22,28^. This leads to local thyroidal dose estimates between 1 Gy for low resolution scintigraphy^23^ and 20 to 80 Gy for micro-SPECT^24,25^, thus making XFI a valuable low-dose alternative.

Using measurements of rat thyroids as a reference^29^, the expected local iodine concentration is in the same order of magnitude as the expected marker concentrations in drug delivery and cell labeling studies^2^, making the thyroid an interesting reference standard for determining the expected sensitivity of XFI for those applications.

While the experiments in this work were performed with sacrificed mice, the measurements have been performed under in-vivo conditions with respect to both, exposure time and radiation dose levels—which are comparable to micro-CT scans—demonstrating the feasibility of in-vivo measurements in the near future.

## Results

The thyroid regions of three different mice were scanned across multiple beamtimes at the P21.1 beamline at the PETRA III synchrotron at DESY, Hamburg, Germany, using a variety of acquisition parameters like spatial resolution and measurement duration. For each mouse, scans with in-vivo conform radiation doses comparable to micro-CT scans as well as high dose reference scans with higher sensitivity where performed. Table 1 summarizes the main scan parameters.

**Table 1:** Summary of the main parameters for the different scans.

Sample	Resolution (mm)	Duration (s)	Photons (per Pixel)	Scan mode
M1	1	1	2.5 × 10^9^	Stepwise
	0.5	10	6.3 × 10^9^	Stepwise
M2	1	1	2.3 × 10^9^	Continuous
	0.2	1	3.4 × 10^9^	Stepwise
M3	1	2	5.4 × 10^9^	Continuous
	0.5	1	1.2 × 10^9^	Continuous
	0.2	5	5.7 × 10^9^	Stepwise

### Quantitative iodine mass reconstruction

**Figure 1:**
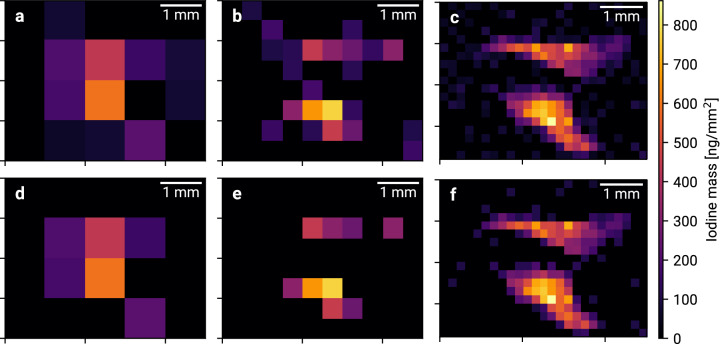
Thyroidal iodine mass maps of mouse M3 for 1 mm, 0.5 mm and 0.2 mm resolution with Z_min_ = 1.6 (**a**–**c**) and Z_min_ = 3 (**d**–**f**).

**Figure 2:**
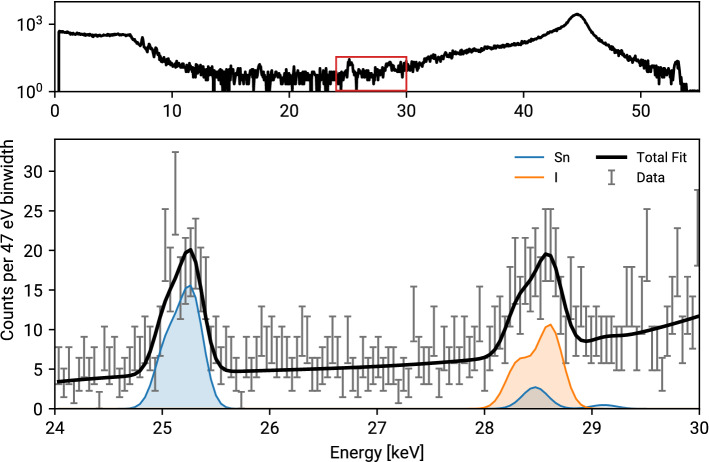
Typical single pixel spectrum (top) and fit of the signal region (bottom) for mouse M3 and 0.2 mm resolution. Detector intrinsic tin fluorescence is present and has to be taken into account for correct iodine reconstruction.

For each scan, the total iodine content as well as the spatial distribution has been reconstructed. Figure 1 shows the iodine distribution of mouse M3 for different beam sizes. For each spectrum, the number of fluorescence photons has been extracted and a hypothesis test for the presence of iodine fluorescence was performed. Two different significance limits Z_min_ are compared. For 1 mm resolution, the thyroid is clearly visible but no further information about its shape can be obtained. At a resolution of 0.5 mm, the two separated thyroid lobes are visible and 0.2 mm resolution reveals the characteristic butterfly shape. Comparing the results for Z_min_ = 3 and Z_min_ = 1.6, a smaller limit yields a higher sensitivity for pixels with less iodine but introduces more noise. A typical single-pixel spectrum and the corresponding fit are shown in Fig. 2.

**Figure 3:**
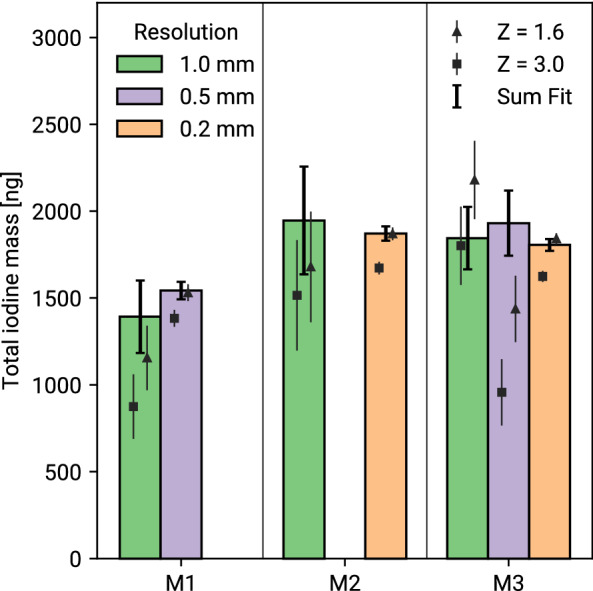
Total reconstructed thyroidal iodine mass for all scans. The bars show the reconstructed mass using the combined spectrum of all pixels, while the triangles and squares show the sum of the reconstructed mass per pixel for Z_min_ = 1.6 and Z_min_ = 3. Only statistical errors are shown. Mean and standard deviation of the combined reconstruction are shown in red.

The total iodine mass in the thyroid has been reconstructed using the sum of the reconstructed masses in the single pixel spectra, as well as for the summed spectrum of all pixels (see “Methods” section for details). The resulting total thyroidal iodine masses are shown in Fig. 3. Using the combined spectra, a good agreement across different mice and resolutions is visible, giving an endogenous thyroid iodine mass of 1.8 ± 0.2 (s.d.) μg.

Pixelwise mass reconstruction yields systematically lower results than using the combined spectrum. This discrepancy increases for lower incident photons per spectrum where more and more pixels are at the edge of the detection threshold while pixelwise and summed reconstruction are consistent for the high statistics scans. The mass reconstruction using the summed spectrum is stable for all dose levels used in our experiments. Comparing the results for Z_min_ = 3 and Z_min_ = 1.6 indicates, that a lower significance threshold can be used when analyzing scans of small regions of interest compared to single spectrum analysis.

### Dose calculation

For all scans, the delivered local dose has been determined using radiochromic films. Additionally, the full body dose of a thyroid scan was estimated. Table 2 lists the full body dose for the different scans as well as the local dose at each single scan position.

**Table 2:** Dose levels and duration of the different scan configurations.

Sample	Resolution (mm)	Full body dose (mGy)	Scan duration (min)	Local dose (mGy)
M1	1	4.4	5	87
	0.5	44.1	80	882
M2	1	4.0	2	80
	0.2	148.8	125	2975
M3	1	8.4	3	168
	0.5	8.4	7	168
	0.2	249.4	291	4987

The estimation of the full body dose and scan duration is based on a scan area of 10 × 10 mm^2^ with an average thickness of 15 mm in the thyroid region, which includes a large tolerance for variation in the thyroid location. If the complete mouse had been scanned, the full body dose would correspond to the local dose. Even for full body scans, the radiation level for low dose scans is comparable to micro-CT scans^30^, and can be performed within the limits of typical anesthesia duration times of up to 60 min. Previous studies have shown, that rodents are capable to recover from a full body dose of about 300 mGy within a few hours^31^, making repeated in-vivo XFI measurements for longitudinal studies feasible.

### Detection limits

Using a 300 mGy local dose limit, 8.6 × 10^9^ incident photons per mm^2^ can be used for in-vivo imaging^32^. An approximation for the lower limit on the detectable iodine mass is given in Eq. (3). For a minimum significance limit Z_min_ = 3 and using Eq. (2), a minimum iodine mass of 120 ng along the 1 mm^2^ wide beam has been determined, corresponding to an average concentration in the beam volume of 8 μg/ml assuming 15 mm thickness of the mouse.

## Discussion

In the present work we have demonstrated an important milestone towards in-vivo XFI—the spatially resolved quantitative measurement of the endogenous iodine mass in murine thyroids, thus, introducing a benchmark for the detection-sensitivity of XFI for small animal studies. We reconstructed a typical endogenous iodine mass of 1.8 μg per thyroid and no considerable difference was found between wildtype and Rag1-deficient mice. The results are consistent between high dose reference scans and low dose in-vivo conform scans. The local radiation dose compared to SPECT/scintigraphy was reduced from 1 to 20 Gy down to 80 mGy and XFI does not require the use of radiotracers.

Furthermore, we demonstrated the feasibility of in-vivo x-ray fluorescence imaging studies for medium-Z elements like iodine down to local concentrations in the order of 8 μg/ml or 120 ng local mass respectively at an in-vivo conform local dose limit of 300 mGy. This is an important milestone for the use of XFI in preclinical studies and paves the way for pharmacokinetic and cell tracking applications^1^. However, further research is needed to verify the expected labeling efficiency and tracer accumulation of labeled cells, especially regarding toxicity as well as cellular- and immune processes. Our present work shows the achievable level of detection sensitivity and from this reference point one can now directly deduce, for future in-vivo cell tracking and pharmacokinetic studies, the minimum local amount of XFI markers required for a measurable signal. The key limiting factor for the sensitivity is the multiple Compton-background. Our chosen incident energy minimizes this background in the spectral region of the iodine signals, enabling the detection of the rather small iodine masses per scan position.

With the current experimental setup, spatially resolved low dose iodine measurements of the thyroid are at the edge of sensitivity limits and can result in systematic underestimation in the total mass reconstruction. This can be overcome by defining a region of interest and combining all obtained spectra in this region, thereby losing the spatial information.

Further improvements towards lower dose or higher sensitivity can easily be achieved by an upgraded experimental setup, mainly increasing the number of detectors. Additionally, silicon drift detectors with larger thicknesses have become commercially available recently^33^, promising to increase the efficiency at 28 keV up to a factor of four. By reducing the footprint of our imaging cell, we expect to be able to halve the detector distance. Combining those aspects, an improvement in sensitivity by a factor of 10 can be achieved in the near future.

## Methods

### Experimental setup

The experiments were performed at the P21.1 beamline at the PETRA III synchrotron at DESY, Hamburg, Germany, delivering about 10^11^ photons/s on a 1 × 1 mm^2^ spot size at 53 keV. The beam size can be adjusted down to 0.1 × 0.1 mm^2^ via a pair of slits and an absorber system can be used to attenuate the beam. The flux is measured by silicon PIN diodes before and after the target area, allowing for a continuous monitoring of the flux and generation of a transmission image.

**Figure 4:**
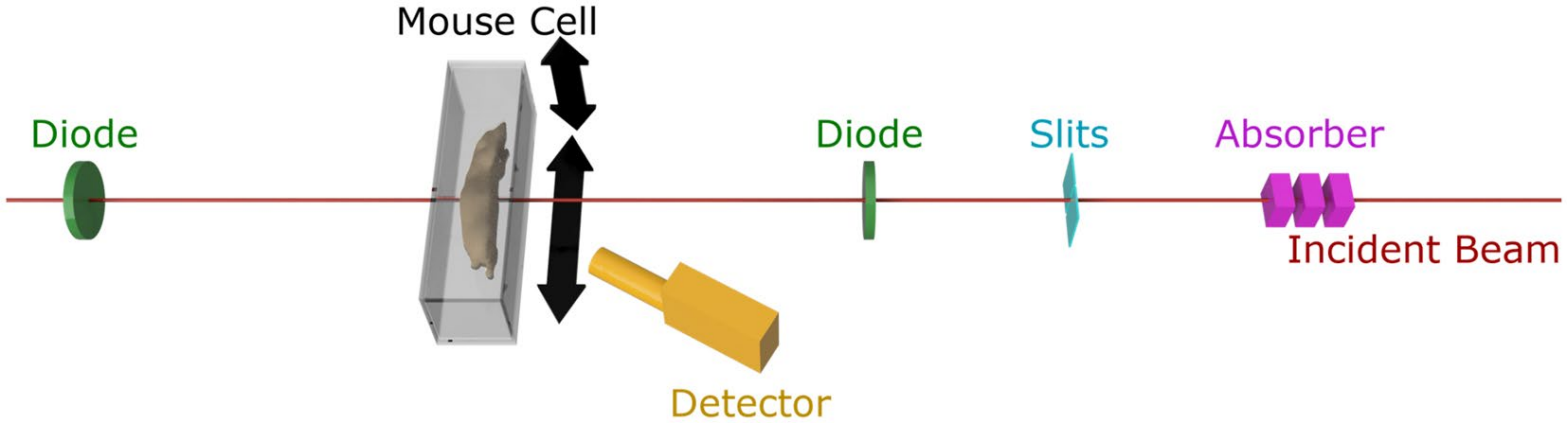
Schematic of the experimental setup. The beam (red) is attenuated by variable absorbers (pink) and collimated using slits (cyan). Its intensity is monitored before and after the interaction region using PIN diodes (green). The SDD (orange) is placed at 150 degree scattering angle in the polarization plane. To minimize attenuation to and from the thyroid, the mouse is lying on the side.

The mouse is placed in a sealed custom-built imaging cell made from 1 mm thick acrylic plates, which is mounted onto a translation arm offering 3 degrees of freedom. A silicon drift detector with a thickness of 0.5 mm and collimated area of 50 mm^2^ (X-123FASTSDD, Amptek Inc, MA, USA) is used for detecting the iodine fluorescence. A schematic of the experimental setup is shown in Fig. 4.

### Detector selection and placement

By using 53 keV incident energy, most of the Compton scattering photons have an energy between 35 and 50 keV—depending on the number of scatter interactions before reaching the detector, hence minimizing their contributions in the K_α_ fluorescence region of iodine at 28.6 keV.

In contrast to x-ray tubes, the incident photons at the P21.1 beamline are horizontally polarized. By placing the detector in the polarization plane, the strong suppression of Compton scattering along this plane as described by the Klein-Nishina cross section can be used for background reduction^34^.

To prevent stress on the cardiovascular system in future in-vivo measurements, the orientation of the mouse was set to be horizontal. Therefore, we did not place the detector at 90° scattering angle, where the minimum background is expected. To reduce the applied dose, the covered solid angle of the detector must be as large as possible, meaning a short distance between target and detector is needed. The minimum distance due to geometric restrictions becomes smaller for scattering angles close to 0° or 180°, where the Compton scattering cross section has its maxima. Therefore, optimum angle and close distance cannot be achieved at the same time. As the applied dose is the most critical aspect, minimizing the detector distance to increase the covered solid angle was prioritized and the detector was placed at 150° scattering angle at a distance of 7 cm to the center of the mouse.

To achieve in-vivo conform scan times of around 60 min, a high incident flux is needed. In our experiments, the maximum usable incident rate is not defined by the beamline flux but the maximum count rate of the detector, limiting the choice of suitable detector materials. While CdTe offers detection efficiency close to 100% in the energy region between 10 and 50 keV, high shaping times are required for an acceptable energy resolution, restricting the count rate to around 100 kcps. Additionally, escape and intrinsic fluorescence events of cadmium and telluride are present in the range between 20 and 30 keV, making it unsuitable for fluorescence measurements of iodine or elements with similar Z.

In contrast, silicon drift detectors (SDDs) offer much better energy resolution and shorter shaping times and can be used with count rates up to 1 Mcps. However, the detection efficiency of the 0.5 mm thick Amptek X-123 SDD at 28 keV is only around 15%, reducing the number of detected fluorescence photons. The efficiency at the maximum of the Compton peak at 45 keV falls off to 4%, resulting in an artificial decrease in the ratio of fluorescence to total count rate and is therefore a desirable feature of silicon-based detectors to minimize scan times.

### Data acquisition

The data acquisition is controlled by a custom software. Hardware interfacing is performed via the Tango control system^35^ which is in use at the beamline. Different scanning modes are available. For high sensitivity measurements with long acquisition times per pixel, a step-based sequential scan is performed. For in-vivo applicable scans with short acquisition times, the motor movements have a large contribution to the total measurement duration and potentially dose if no fast shutter is used to block the beam during motor movements. Therefore, an alternative scan mode is available, in which the horizontal axis is translated continuously, and spectra are recorded on the fly. In this mode, the time and dose overhead introduced by the travel intervals is removed. However, due to the sliding window of exposure along the sample, this introduces some overlap between adjacent pixels. Each mouse was scanned at least once with a high resolution and high dose scan, providing a reference measurement and once with an in-vivo conform low dose scan. For M2 and M3, this scan was performed in continuous mode, while the M1 low dose scan was done in the step-based mode to demonstrate the reduction of the overall measurement duration in continuous mode.

For each acquisition point, the detector spectrum, the diode current and meta-information like motor positions are recorded. For step-based scans, a quadratic beam shape of 0.5 mm × 0.5 mm or 0.2 mm × 0.2 mm is selected and the mouse is translated by the beamwidth for each step. In continuous mode, a narrow beam – e.g. 0.2 mm × 1 mm—with the short side along the continuous axis is used to minimize blurring between neighboring datapoints. The spectrum is recorded over a travel length of 1 mm and the measurement duration per point is controlled by changing the motor speed.

While 3D imaging such as x-ray fluorescence computed tomography allows for better separation of organs, it requires a three-dimensional scanning including a rotational axis with typically between 30 and 45 steps^10,13,15^. For the same radiation dose, this reduces the overall sensitivity since the statistics per measurement are lower and results in an increased total acquisition time due to additional motor movements. As we focus on maximizing the sensitivity, we have opted for a 2D scan which still offers a good separation of most organs.

### Data analysis

Each spectrum is normalized to the average incident flux during the scan. For accurate signal separation, peak fitting has to be performed^36^. To extract the number of detected fluorescence photons, a fit model based on the ratio of tabulated fluorescence cross sections is used^37^. For each existing fluorescence line of an element in the fit region, a Gaussian with a relative height corresponding to the cross section of this transition adjusted by the detection efficiency at this energy is added to the fit model. The width of the Gaussian is fixed and based on the measured detector resolution. A single fit parameter per element for the absolute scaling of its combined fluorescence spectrum is then used to model the data. Additionally, a 3rd order polynomial is added to the fit to model the continuous Compton background contribution.

For an accurate fit of the iodine fluorescence, the fluorescence lines of tin must be added to the model. It is present in the detector and excited by the high energy Compton photons and therefore visible in the recorded spectrum. Tin has its K_β_ fluorescence energy at 28.4 keV and thus lies within the energy resolution of the iodine K_α_ line. Using the tin K_α_ peak height and the efficiency corrected K_α_/K_β_ ratio, tin contributions in the iodine region can be inferred.

Assuming no attenuation of both the incident and the fluorescence photons, the iodine mass $M_{F}$ at each datapoint can be calculated from the number of detected fluorescence photons using

1 $$\begin{array}{*{20}c} {M_{F} = \frac{{N_{F} }}{{N_{0} }} \cdot \frac{{A_{B} }}{{\sigma_{F} \varepsilon_{F} }} \cdot \frac{{4\pi r_{D}^{2} }}{{A_{D} }}} \\ \end{array}$$

with $N_{F}$ the number of detected fluorescence photons, $N_{0}$ the number of incident photons, $A_{B}$ the area covered by the beam, $\sigma_{F}$ the fluorescence cross section, $\varepsilon_{F}$ the detector efficiency at the fluorescence energy, $r_{D}$ the detector distance and $A_{D}$ the detector surface area. Cross section and efficiency values are obtained using the xraylib library^37^.

Attenuation effects can be neglected, as the thyroid is closely behind the skin with very little tissue in the beam path. Assuming a maximum tissue thickness of 3 mm in front of the thyroid, the expected total attenuation is about 15%.

For each fit a single sided hypothesis test is performed. Only spectra with statistically significant iodine fits are contributing to the reconstructed mass. Two different lower significance limits—3σ and 1.6σ, resulting in a probability for false positives of less than 0.15% and 10% per pixel (see^9^ for details)—are applied to compare the tradeoff between sensitivity and specificity.

However, this method introduces systematic underestimation of the total mass when the single pixel spectra are close to the detection threshold. Alternatively, the combined spectrum of all pixels in the thyroid region can be used for the calculation of the total mass and therefore reducing statistical noise at the cost of losing the spatial information. Both methods are performed and compared.

### Detection limit estimation

For small changes in the detector position, the number of background photons $N_{B}$ in the signal energy range can be approximated by

2 $$\begin{array}{*{20}c} {N_{B} = R_{B} N_{0} \frac{{A_{D} }}{{4\pi r_{D}^{2} }} } \\ \end{array}$$

where $R_{B}$ describes the ratio of photons created by scattering contributing to the background in the signal region and the total number of incident photons.

The significance of a signal can be approximated via $Z \approx N_{F} /\sqrt {N_{B} }$^38^. Equations (1) and (2) can be used to obtain a minimum detectable mass covered by the beam via

3 $$\begin{array}{*{20}c} {\frac{{M_{min} }}{{A_{B} }} = \frac{{Z_{min} }}{{\sigma_{F} \sqrt {\varepsilon_{F} } }}\sqrt {\frac{{R_{B} }}{{N_{0} }}\frac{{4\pi r_{D}^{2} }}{{A_{D} }}} } \\ \end{array} .$$

The key to increasing the sensitivity is thus to minimize $R_{B}$ by choosing an optimum incident photon energy and detector angle where the background contribution $R_{B}$ is small while at the same time the fluorescence cross section, which falls off for higher energies, is still large.

The fraction of incident photons contributing to the detected background in the signal region $R_{B}$ has been experimentally determined to be 6 × 10^–4^ for our setup and the detection efficiency for iodine K_α_ fluorescence is $\varepsilon_{F}$ = 0.15. At 53 keV incident energy, the iodine K_α_ fluorescence cross section is $\sigma_{F}$ = 6.1 cm^2^/g.

### Incident flux and dose measurement

During the measurements, the relative photon flux both before and after the sample is monitored using silicon PIN diodes. Before starting the mouse scans, thin film fluorescence reference targets with precisely known thicknesses of RbI and Ag (Micromatter Technologies Inc, Canada) were used for absolute flux measurements and calibration of the diode current by using Eq. (1) to obtain N_0_. Additionally, a series of exposures of radiochromic films (Gafchromic EBT3, Ashland Advanced Materials, NJ, USA) was used to obtain a conversion of diode current to air-kerma rate by irradiating the film free in air at the center of the target area. The radiochromic films were calibrated in solid water using a 6 MV radiotherapy accelerator at the Department of Radiotherapy and Radiation Oncology, University Medical Center Hamburg-Eppendorf and no significant dose sensitivity difference compared to 53 keV x-rays is expected^39^.

Using these two calibrations, it is possible to continuously monitor both dose and absolute number of photons during the whole experiment.

For 53 keV incident energy and a tissue thickness of around 15 mm, corresponding to the thickness of the mice in the thyroid region, the expected local dose rate is about 350 mGy/(10^10^ photons/mm^2^)^32^. This is in good agreement with the assessment using the reference targets and radiochromic films, obtaining an air-kerma rate of 320 mGy/(10^10^ photons/mm^2^) and a conversion rate of about 1.1 from air-kerma to dose in mouse-sized phantoms at 53 keV^39^.

### Animal studies

Two C57BL/6 Rag1-deficient (12 weeks and 16 weeks old) mice and one wild-type C57BL/6 (28 weeks old) mouse were selected based on availability. The mice were fed with a normal chow diet. No significant effect on thyroidal iodine is expected from the gender or age difference^41–43^.

All mice were cared for in accordance with German animal ethics regulations and experimental protocols were approved by the institutional review board ‘Behörde Justiz und Verbraucherschutz’, Hamburg, Germany (number ORG_998 and N 024_20). Mice were kept under specific pathogen free conditions, ambient temperature of 20 ± 2 °C, humidity of 55 ± 10% and a dark/light cycle of 12 h. The experiments were performed in accordance with the ARRIVE guidelines.

## Data Availability

All raw and processed data generated in this work are available from the corresponding author on reasonable request.
